# Non-Structural Protein NSm of *Tomato Spotted Wilt Virus* Is an Avirulence Factor Recognized by Resistance Genes of Tobacco and Tomato via Different Elicitor Active Sites

**DOI:** 10.3390/v10110660

**Published:** 2018-11-21

**Authors:** Changjun Huang, Yong Liu, Haiqin Yu, Cheng Yuan, Jianmin Zeng, Lu Zhao, Zhijun Tong, Xiaorong Tao

**Affiliations:** 1 Yunnan Academy of Tobacco Agricultural Sciences, Key Laboratory of Tobacco Biotechnological Breeding, National Tobacco Genetic Engineering Research Center, Kunming 650021, China; Yong2liu@sina.com (Y.L.); yuhaiqin@hotmail.com (H.Y.); craig.cy@foxmail.com (C.Y.); zengjm2017@163.com (J.Z.); zhaolu66@outlook.com (L.Z.); tzj861@163.com (Z.T.); 2 Key Laboratory of Integrated Management of Crop Diseases and Pests, Ministry of Education, Department of Plant Pathology, Nanjing Agricultural University, Nanjing 210095, China

**Keywords:** *Tomato spotted wilt virus*, hypersensitive response, avirulence factor, movement protein

## Abstract

*Tomato spotted wilt virus* (TSWV) is one of the most destructive viral pathogens of plants. Recently, a single dominant gene conferring complete resistance to TSWV (RTSW) was identified in *Nicotina alata* and introgressed into cultivated tobacco (*N. tabacum*). However, whether the TSWV carries an avirulence (Avr) factor directed against RTSW remains obscure. In the present study, we identified the non-structural protein (NSm), the movement protein of TSWV, which is an RTSW-specific Avr factor, by using two different transient expression systems. Using amino acid (aa) substitution mutants, we demonstrated the ability to induce RTSW-mediated hypersensitive response (HR) of NSm is independent of its movement function. Moreover, key substitutions (C118Y and T120N), a 21-aa viral effector epitope, and different truncated versions of NSm, which are responsible for the recognition of the *Sw-5b* resistance gene of tomato, were tested for their ability to trigger HR to TSWV in tobacco. Together, our results demonstrated that RTSW-mediated resistance is triggered by NSm in the same way as by Sw-5b, however, via different elicitor active sites. Finally, an *Avr* gene-based diagnostic approach was established and used to determine the presence and effectiveness of resistance genes in tobacco.

## 1. Introduction

According to the zigzag model of plant–microbe interactions [1], the plant’s innate immune system is broadly divided into two different layers: pathogen-associated molecular pattern (PAMP)-triggered immunity (PTI) and effector-triggered immunity (ETI). PTI is activated by specific recognition between PAMPs, such as bacterial flagellin and fungal chitin, and the corresponding membrane-anchored pattern recognition receptors (PRRs) of plants, which serve as the first line of defense against invading pathogens. To overcome PPRs, pathogens encode virulence factors (effectors) that interfere with PTI and enable them to activate effector-triggered susceptibility (ETS) [2,3]. To counteract ETS, plants have evolved intracellular resistance (R) proteins, which directly or indirectly recognize pathogen effectors or avirulence (Avr) factors and activate ETI, which is often manifested in the form of a hypersensitive response (HR), characterized by rapid cell death, the production of reactive oxygen species (ROS) and salicylic acid (SA), and expression of defense-related genes [1,4]. Recently, several lines of evidence have shown that virus-derived PAMPs are also capable of activating PTI-like responses. However, more and more R proteins and Avr factors characterized in different viruses substantiate the notion that plants deploy the innate immune system to fight viruses in a typical ETI-based manner [4].

In the past few decades, the unveiling of whole genome sequences of plants has facilitated the genome-wide identification of disease resistance genes, revealing that plants possess a large number of R genes. Most R genes in plants belong to the nucleotide-binding site leucine-rich repeat (NLR, also known as NB-LRR) superfamily. In the family Solanaceae, 326, 755, and 755 NLR-encoding genes have been identified in tomato (*Solanum lycopersicum*), potato (*S. tuberosum*), and pepper (*Capsicum annuum*), respectively [5,6,7]. To date, more than 100 R genes, providing resistance to various pathogens, have been cloned in any given host. However, only a small number of R genes have been characterized in different plant species, including the model plant *Arabidopsis thaliana* [8]. Because a functional R protein can be identified only in conjunction with its Avr counterpart, one of the ways to characterize an R gene is through the identification of its Avr target.

*Tomato spotted wilt virus* (TSWV), a type member of the order Bunyavirales, family Tospoviridae, and genus *Orthotospovirus*, is distributed worldwide, and causes severe economic losses in many crops [9,10,11]. The TSWV virions are spherical membrane-bound particles of 80–120 nm diameter, and contain a tripartite single-strand (ss) RNA genome composed of large (L), medium (M), and small (S) segments, depending on their size. The TSWV genome encodes for four structural and two non-structural proteins. The L segment has a negative polarity, and encodes RNA-dependent RNA polymerase (RdRp) on the viral complementary (vc) RNA. The M segment has ambisense polarity, and encodes the precursors of two glycoproteins (Gn and Gc) and a non-structural protein (NSm) on the vc and viral (v) RNA, respectively. The S segment has ambisense polarity, and encodes the nucleocapsid (N) protein and non-structural protein NSs on vc and v RNAs, respectively [10,11].

TSWV is one of the most destructive viral plant pathogen, ranking second among the most important plant viruses worldwide [12]. Moreover, TSWV has a very wide host range and infects more than 1000 plant species, including many important vegetables, legumes, ornamental crops, and weeds [13]. In nature, TSWV is transmitted in a circulative-propagative manner exclusively by thrips, which are extremely difficult to control because of their small size, rapid developmental time, high reproductive rate, and insecticide resistance. Because of the broad host range of TSWV and difficult to control vector, TSWV is a significant threat to agricultural crops [10]. To minimize the damage caused by TSWV and to avoid excessive use of insecticides, the identification of natural R genes is an attractive approach [11]. To date, only two single-dominant R genes have been cloned: Sw-5b in tomato and Tsw in pepper; both genes are well characterized and are available for commercial resistance breeding [14,15,16]. Additionally, both Sw-5b and Tsw belong to the nucleotide-binding site leucine-rich repeat (NBS-LRR) family of R genes, and recognize TSWV proteins to induce pathogen resistance in plant hosts; Sw-5b recognizes NSm [17,18], and Tsw recognizes NSs [19].

In 1969, Opoka reported a wild tobacco species, *Nicotiana alata*, that was completely resistant to TSWV [20]. The locus conferring resistance to TSWV, hereafter referred to as *RTSW*, was introgressed into cultivated tobacco (*N. tabacum* L.) using *N. otophora* as a bridging parent, resulting in the breeding line “Polalta” [21]. The segregation ratio of the F2 population derived from the *N. tabacum* × Polalta cross indicates that RTSW is a single dominant gene conferring resistance to TSWV [22,23]. Although several TSWV resistance markers have been developed, they have neither been mapped nor isolated. Therefore, the molecular mechanism of RTSW-mediated resistance to TSWV remains unknown [23]. Furthermore, a corresponding viral component that acts as an Avr factor against RTSW has not been identified.

In the present study, we show that the NSm protein of TSWV acts as the Avr determinant of RTSW-based resistance using two different transient expression systems. Moreover, we show that intercellular trafficking of NSm is uncoupled from its function in HR induction, and the elicitor active sites (EASs) of NSm are unique for RTSW and Sw-5b.

## 2. Materials and Methods

### 2.1. Plasmid Construction

Total RNA was isolated from TSWV infected *Nicotiana benthamiana* plants. To generate transient expression vectors of TSWV, five full-length open reading frames (ORFs) of genes encoding Gn, Gc, NSm, N, and NSs proteins were amplified from total RNA by reverse transcription PCR (RT-PCR), using gene-specific primer pairs (NSmBamHIF/NSmBamHIR, GnBamHIF/GnBamHIR, GcBamHIF/GcBamHIR, NSsBamHIF/NSsBamHIR, and NBamHIF/NBamHIR) (Table A1). The PCR products were digested with *Bam*HI and individually cloned into *Bam*HI-digested p2300-35S vector between the *Cauliflower mosaic virus* (CaMV) 35S promoter and 35S terminator. The remaining constructs, including p2300-35S-Sw-5b, p2300-35S-Sm1-248 (NSm1-248aa), p2300-35S-NSm1-195 (NSm1-195aa), p2300-35S-NSm51-303 (NSm51-303aa), and p2300-35S-NSm101-303 (NSm101-303aa), were constructed as described recently [16].

To assess the pathogenicity of NSm in *Nicotiana* species, the NSm ORF was amplified by PCR using the primer pair NSmCalIF/NSmSalIR and inserted into the pGR107 vector [24] to produce the potato virus X (PVX)-NSm construct.

Wild type and mutant versions of the NSm gene were fused with the yellow fluorescent protein (YFP) gene to generate C-terminal fusions of YFP (NSm-YFP, NSmC118Y-YFP, NSmT120N-YFP, NSm115-135aa-YFP, NSmA54–56-YFP, NSmA93–94-YFP, NSmA122–125-YFP, NSmA154-YFP, and NSmA269–274-YFP) as described previously [16,25].

### 2.2. Plant Materials, Virus Inoculation, and Agroinfiltration

TSWV was isolated from local diseased plants in the Yunnan province of China, and propagated on *N. benthamiana* plants [26]. At the 4–6-leaf stage, *N. benthamiana* plants were rub-inoculated with silicon dioxide and virus-containing sap at 1 month after planting, as described previously [27].

Different constructs were individually mobilized into *Agrobacterium tumefaciens* strain EHA105 via electroporation, and the transformed agrobacteria were grown overnight in Luria-Bertani (LB) media containing 10 mM 2-(N-morpholino) ethanesulphonic acid (MES), 20 μM acetosyringone, and appropriate antibiotics. Before agroinfiltration, suspensions of transformed agrobacteria were adjusted to an optical density (OD_600_) of 0.7 in MES buffer (10 mM MgCl_2_, 10 mM MES [pH 5.6], and 150 μM acetosyringone), and incubated at room temperature for 2–4 h. To analyze the transient expression of wild-type and mutant NSm, cultures were infiltrated into the abaxial surface of leaves using a 1 mL syringe. For agroinfection of PVX derivatives, agrobacteria carrying individual recombinant full-length cDNA clones of PVX were used to infiltrate tobacco plants.

Virus-inoculated and agrobacteria-infiltrated tobacco plants were grown in an insect-free growth chamber at 25 °C under a 14-h light/10-h dark photoperiod, and observed daily for symptom development.

### 2.3. RNA Isolation, RT-PCR, and Quantitative Real-time PCR (qRT-PCR)

Leaves were harvested from plants infected with TSWV or infiltrated with *A. tumefaciens* cultures harboring different constructs, and ground in liquid nitrogen. Total RNA was extracted from leaves, and first-strand cDNA was synthesized using PrimeScript First Strand cDNA Synthesis Kit (TaKaRa, Dalian, China). RT-PCR was used to check NSm gene expression following inoculation with TSWV. Plants with no obvious symptoms and no NSm expression were classified as resistant, and the others were designated as susceptible.

A qRT-PCR assay was performed on the LightCycler 480^@^ II machine with LightCycler 480^@^ SYBR I Master PCR Mix (Roche Applied Science, Basel, Switzerland). Thermocycling conditions were as follows: 95 °C for 5 min, followed by 40 cycles of 95 °C for 10 s, 60 °C for 15 s, and 72 °C for 20 s, and lastly, a melting curve analysis (68 °C to 95 °C). Primers used in the qRT-PCR assay were designed using the Premier 6 (Premier-Biosoft, Palo Alto, CA, USA) software (Table A1). The glyceraldehyde-3-phosphate dehydrogenase (*GAPDH*) gene was used as an internal control to normalize gene expression data. Six biological and three technical replicates were performed for each experiment, to minimize biological variation and random noise associated with the equipment.

### 2.4. DAB Staining

The accumulation of H_2_O_2_ was visually detected in leaves using 3,3-diaminobenzidine (DAB) staining, as described previously [28], with minor modifications. Briefly, entire leaves were excised at the base of the petiole and incubated in DAB solution (1 mg/mL; pH 3–3.8) (Sigma-Aldrich, St Louis, MO, USA) for 8 h at 25 °C. Subsequently, leaves were immersed in boiling ethanol (96%) for 5–10 min, cooled, preserved at room temperature in 70% ethanol, and photographed.

### 2.5. Protein Extraction and Western Blot Analysis

Leaves of tobacco plants infiltrated with *A. tumefaciens* cultures harboring different constructs were ground in liquid nitrogen, and 100 mg of powder was mixed with 100 μL of 2 × sodium dodecyl sulfate (SDS) sample buffer. Total extracts were boiled for 5 min and then chilled on ice for 3 min. Samples were then centrifuged for 5 min at 13,000 × *g*. Proteins were separated via SDS-polyacrylamide gel electrophoresis (PAGE) on 12.5% polyacrylamide gels and transferred to nitrocellulose membranes. The membranes were probed with rabbit anti-green fluorescent protein (GFP) polyclonal antibody (ab6556, Abcam, Cambridge, MA, USA), followed by a goat anti-rabbit immunoglobulin G (IgG) conjugated with horseradish peroxidase (HRP) (ab6721, Abcam) as the secondary antibody. Membranes were incubated in working solution of ECL at room temperature for 2 min (Cwbio, Jiangsu, China), and the signal was measured with an Amersham Imager 680 (GE, San Francisco, CA, USA).

### 2.6. Enzyme-linked Immunosorbent Assay (ELISA)

Systemic leaves of *N. benthamiana* infected with TSWV, PVX, or PVX-NSm were ground and coated overnight in enzyme-linked immunosorbent assay (ELISA) plates, as described previously [29]. The coated samples were incubated for 1 h in mouse monoclonal antibody (1:5000 dilution) produced from the purified virus. Subsequently, goat anti-mouse IgG (1:8000 dilution) conjugated with alkaline phosphatase was applied to the wells, and incubated for 1 h. After 30-min incubation in NBT-BCIP solution (Promega, Madison, WI, USA), ELISA absorbance values were measured using a Microplate Reader Model 680 (Bio-Rad, Hercules, California, USA).

## 3. Results

### 3.1. Tobacco RTSW Confers Resistance to TSWV by a Hypersensitive Response (HR)

**Figure 1:**
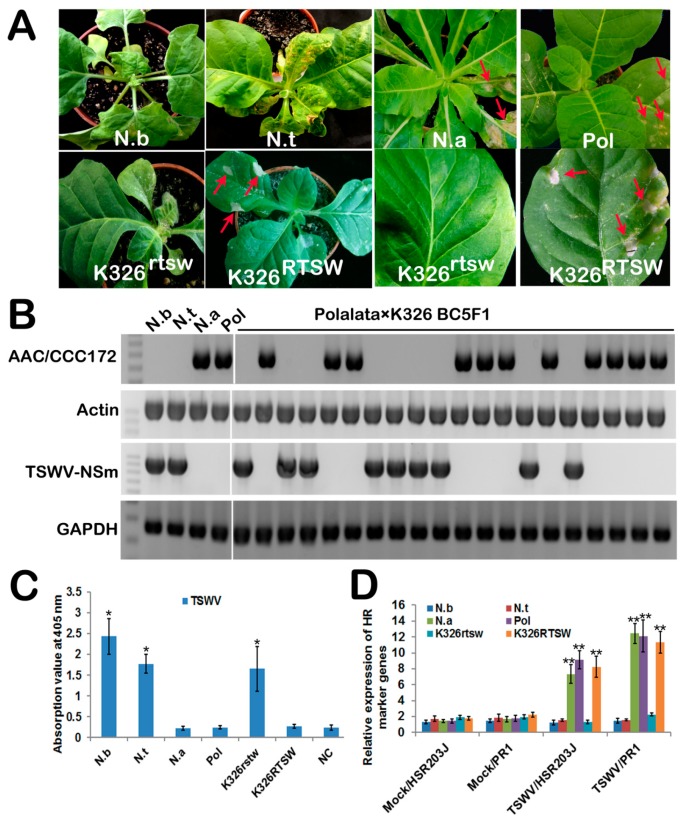
Tobacco plants harboring the resistance to *Tomato spotted wilt virus* (RTSW) gene demonstrate resistance to the *Tomato spotted wilt virus* (TSWV) via a hypersensitive response (HR). (**A**) Phenotype of tobacco leaves inoculated with TSWV. N.b, *Nicotiana benthamiana*; N.t, *N. tabacum* cv. K326; N.a, *N. alata*; Pol, Polalta; K326^rtsw^, plants derived from the backcross segregating (BC5F1) population of Polalta × K326 cross lacking the RTSW gene and susceptible to TSWV; K326^RTSW^, plants derived from the same BC5F1 population but carrying the RTSW gene and are hence resistant to TSWV. Photographs of plants were taken at 14 days post-inoculation (dpi). Bottom two panels on the right show enlarged views of K326^rtsw^ and K326^RTSW^ leaves. Red arrows indicate HR symptoms in inoculated leaves. (**B**) Genotyping using SCAR markers S-AAC/CCC172 linked with the TSWV resistance gene (top two panels), and reverse transcription-polymerase chain reaction (RT-PCR) analysis of systemic infection of TSWV (bottom two panels) in tobacco plants. For RT-PCR, RNA of un-inoculated upper leaves was used as a template for the non-structural protein (NSm) gene amplification at 14 dpi. Actin and glyceraldehyde-3-phosphate dehydrogenase (GAPDH) genes were used as an internal control in genotyping and RT-PCR, respectively. (**C**) Enzyme-linked immunosorbent assay (ELISA) of TSWV titer in systemic leaves shown in panel (A). Data represent mean ± standard deviation (SD) (*n* = 6). Un-inoculated leaves of wild-type *N. tabacum* plants were used as a negative control (NC). Statistically significant differences are indicated with an asterisk (*, *p* < 0.05; Student’s *t*-test). (**D**) Quantitative real-time PCR (qRT-PCR) analysis of HSR203J and pathogenesis-related 1 (PR1) gene expression in systemic leaves shown in panel (A). The GAPDH gene was used as a reference. Data represent mean ± standard error of the mean (SEM) of three independent experiments (*n* = 6). Statistically significant differences are indicated with asterisks (**, *p* < 0.01; Student’s *t*-test).

To clarify the characteristics of *RTSW*-mediated resistance to TSWV, we inoculated leaves of *N. alata*, Polalta, *N. benthamiana*, and *N. tabacum* cv. K326 with TSWV; *N. alata* and Polalta carry the *RTSW* gene, whereas *N. benthamiana* and K326 lack this gene. The susceptible genotypes (*N. benthamiana* and K326) developed typical orthotospovirus-like symptoms on systemic leaves at 10–14 days post inoculation (dpi). Systemic infected leaf tissues showed chlorosis, necrotic spots, and deformation (Figure 1A), and eventually turned necrotic; new shoots withered or died, and petioles and stems collapsed. However, *N. alata* and Polalta plants showed a resistant phenotype, characterized by a local HR, i.e., development of necrotic and chlorotic spots on TSWV-inoculated leaves at 3 dpi but no symptoms on systemic leaves during the entire growth period (Figure 1A). To rule out host-specific effects on the resistance phenotype, 20 plants from the backcross segregating population (BC5F1) derived from the Polalta × K326 cross were randomly selected and mechanically inoculated with TSWV. These plants were genotyped with a previously reported sequence characterized amplified region (SCAR) marker, S-AAC/CCC172 [23]. Results showed that 11 of the 20 plants were positive (K326^RTSW^), and the remaining 9 were negative (K326^rtsw^) for the RTSW gene; thus, the ratio of resistant to susceptible plants was approximately 1:1, as expected for the segregation of a single dominant gene (Figure 1B). All K326^rtsw^ plants inoculated with TSWV developed typical orthotospovirus-like symptoms on systemic leaves at 10–14 dpi, similar to the susceptible genotypes, K326 and *N. benthamiana*. However, all K326^RTSW^ plants exhibited HR only on the inoculated leaves at 3 dpi, and showed no visible symptoms on systemic leaves during the entire growth period (Figure 1A). To investigate the absence of symptoms on systemic leaves, systemic leaf samples were collected from TSWV-inoculated plants, and virus accumulation was analyzed using ELISA and RT-PCR. Our results showed that TSWV was not present in un-inoculated upper systemic leaves of *N. alata*, Polalta, and K326^RTSW^ plants (Figure 1B and C).

Next, we examined the expression of two well-characterized HR marker genes, HSR203J and pathogenesis-related 1 (PR1) [30], in the leaves of susceptible and resistant genotypes at 3 dpi using qRT-PCR. The results showed that both HSR203J and PR1 genes were specifically and dramatically up-regulated in *N. alata*, Polalta, and K326^RTSW^ leaves inoculated with TSWV compared with mock-inoculated leaves or TSWV-inoculated leaves of *N. benthamiana*, K326 and K326^rtsw^, all of which showed similarly low levels of expression of HSR203J and PR1 genes (Figure 1D).

### 3.2. NSm Protein is an Elicitor of RTSW-based Resistance to TSWV

**Figure 2:**
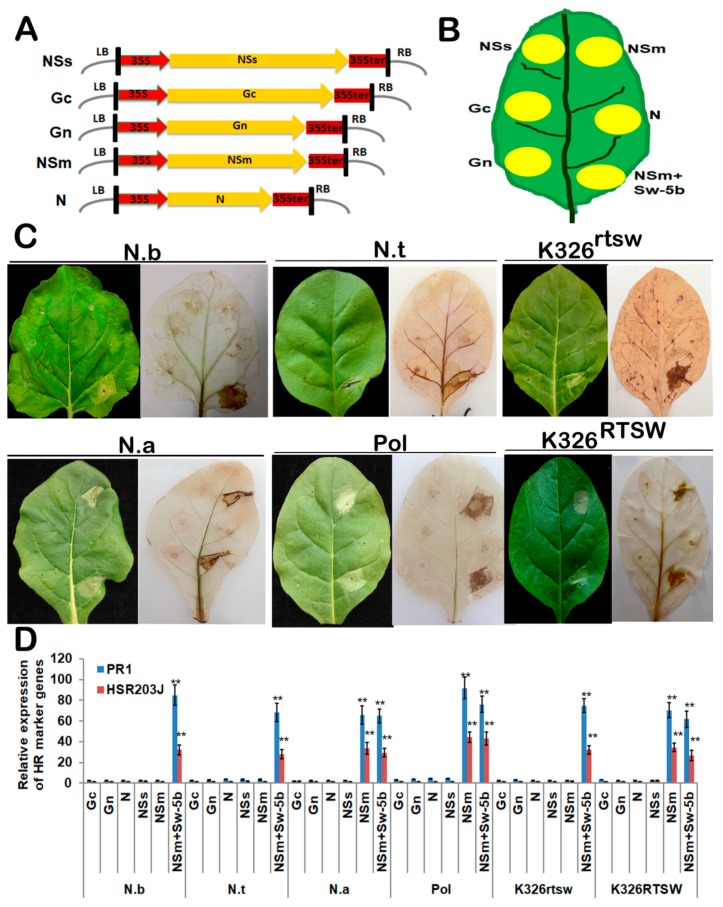
Transient expression of TSWV genes using agroinfiltration of 35S-based binary vectors in tobacco leaves. (**A**) Schematic representation of 35S-based expression cassettes. Five open reading frames (ORFs) were inserted between the 35S promoter (35S) and 35S terminator (35Ster). LB, left border; RB, right border. (**B**) Schematic representation of a tobacco leaf showing the infiltration areas of various 35S-based expression cassettes. (**C**) Photographs of tobacco leaves exhibiting HR symptoms (left) or production of H_2_O_2_ (right) 2 days after agroinfiltration with transient expression constructs. Leaves on the right were stained with 3,3-diaminobenzidine (DAB). Co-infiltration of NSm with Sw-5b (NSm + Sw-5b) was used as a positive control in each leaf. N.b, *Nicotiana benthamiana*; N.t, *N. tabacum* cv. K326; N.a, *N. alata*; Pol, Polalta; K326^rtsw^, plants derived from the BC5F1 population of Polalta × K326 lacking the *RTSW* gene and susceptible to TSWV; K326^RTSW^, plants derived from the sme BC5F1 population but carrying the RTSW gene and resistant to TSWV. (**D**) qRT-PCR analysis of HSR203J and PR1 expression in leaves shown in panel (C). The GAPDH gene was used as a reference. Data represent mean ± SEM of three independent experiments (*n* = 6). Asterisks indicate statistically significant differences (**, *p* < 0.01; Student’s *t*-test).

To identify the TSWV gene(s) responsible for the induction of HR phenotype in plants, we expressed the genes encoding Gn, Gc, NSm, N, and NSs proteins under the control of the CaMV 35S promoter (Figure 2A). These expression cassettes were mobilized into *A. tumefaciens*, and agroinfiltrated into the leaves of *N. alata*, Polalta, *N. benthamiana, N. tabacum* cv. K326, K326^RTSW^ and K326^rtsw^ (Figure 2B). Agroinfiltration of constructs expressing NSm and positive control (NSm+Sw-5b) elicited necrotic local lesions in *N. alata*, Polalta, and K326^RTSW^ leaves, resembling HR-type cell death, within 2 dpi (Figure 2C), with the infiltrated tissues becoming completely necrotic by 3 dpi. By contrast, only the positive control construct elicited HR-type cell death in the leaves of *N. benthamiana*, K326, and K326^rtsw^ (Figure 2C).

The production of ROS in agroinfiltrated leaves was assayed by in situ detection using DAB staining. In contrast to *N. benthamiana*, K326 and K326^rtsw^ leaves only infiltrated with the positive control construct turned brown at 2 dpi (Figure 2C). Levels of hydrogen peroxide (H_2_O_2_) in leaves of *N. alata*, Polalta and K326^RTSW^ expressing the NSm gene were similar to those in leaves expressing the positive control construct (Figure 2C), implying that the *NSm* gene triggers H_2_O_2_ accumulation in tobacco genotypes carrying the RTSW gene.

To further determine whether NSm-induced necrosis comprises HR-type cell death, leaf areas infiltrated with different constructs were collected, and the expression of HSR203J and PR1 was analyzed by qRT-PCR at 24 hours post-inoculation. The results showed that HSR203J and PR1 genes were significantly induced in *N. alata*, Polalta, and K326^RTSW^ leaves inoculated with NSm, whereas their expression was similarly low in leaves infiltrated with other TSWV genes or leaves of *N. benthamiana*, K326, and K326^rtsw^ tobacco plants infiltrated with NSm (Figure 2D).

### 3.3. TSWV NSm Elicits HR-type Cell Death in a PVX Expression Assay Against RTSW Background

**Figure 3:**
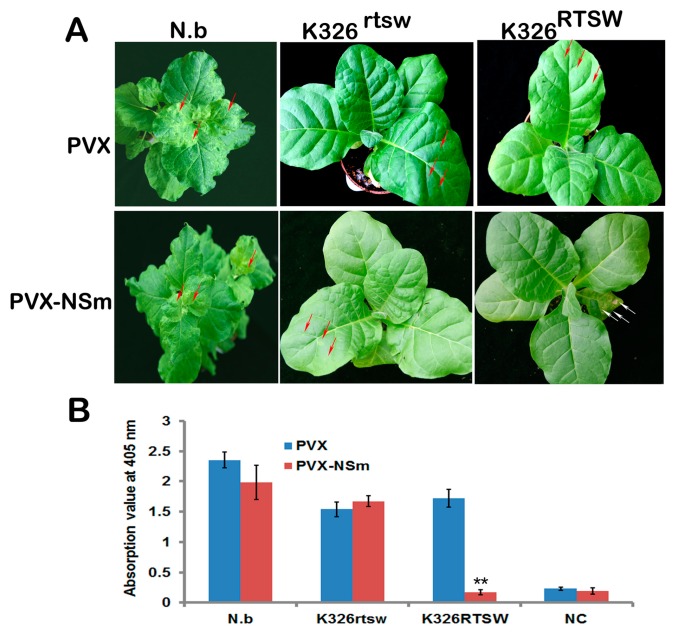
Transient expression of TSWV NSm using argoinfection of *Potato virus X* (PVX)-based vectors. (**A**) Phenotypes of tobacco leaves were agroinfiltrated with PVX or PVX-NSm constructs. Photographs were taken at 12 dpi. PVX-induced faint chlorotic spots are indicated with red arrows, and HR symptoms in inoculated leaves are indicated with white arrows. N.b, *Nicotiana benthamiana*; K326^rtsw^, plants derived from the BC5F1 population of Polalta × K326 lacking the RTSW gene and susceptible to TSWV; K326^RTSW^, plants derived from the same BC5F1 population, but carrying the RTSW gene and resistance to TSWV. (**B**) ELISA of PVX titer in systemic leaves shown in panel (A). Data represent mean ± SD in one representative experiment (*n* = 6). Un-inoculated healthy *N. benthamiana* plants served as a negative control (NC). Asterisks indicate statistically significant differences (**, *p* < 0.01; Student’s *t*-test).

Previous evidence has shown that several elicitors of HR-type cell death prevent systemic infection, resulting in pathogen arrest [31]. Because of the lack of an infectious TSWV clone, we used the heterologous PVX model system, which has been used for the systemic ectopic expression of the cloned gene [24]. The NSm gene was cloned into the PVX vector pGR107 for agroinfiltration into the leaves of *N. benthamiana*, K326^RTSW^, and K326^rtsw^ (Figure 3A). An empty vector was used as a control. Typical PVX-like symptoms such as veinal chlorosis and subsequent systemic mild chlorotic symptoms were observed in *N. benthamiana* at 7 dpi (Figure 3B) and viral RNAs from both PVX constructs were detected in *N. benthamiana* using RT-PCR. Six plants each of K326^RTSW^ and K326^rtsw^ were agroinfiltrated with PVX and PVX-NSm. Although both vectors were equally expressed, repeated analysis consistently revealed a dark brownish necrosis, typical of an HR-like response, only in K326^RTSW^ leaves agroinfected with PVX-NSm (Figure 3A). Remarkably, PVX-NSm elicited necrotic lesions on all inoculated leaves of K326^RTSW^, as early as 3–4 dpi; however, no visible symptoms were observed in systemic leaves throughout the experiment. By contrast, K326^RTSW^ plants infiltrated with PVX, or K326^rtsw^ plants infiltrated with both vectors initially showed only mild symptoms on inoculated leaves, characterized by a faint systemic chlorosis at 7–9 dpi, followed by the appearance of mild chlorotic spots (Figure 3A). Furthermore, ELISA experiments using a PVX monoclonal antibody revealed the presence of PVX in systemic leaves of all plants infected with PVX, and specifically, in K326^rtsw^ plants infected with PVX-NSm, though not in K326^RTSW^ leaves infected with PVX-NSm (Figure 3B). Thus, these results suggest that NSm plays a critical role in inducing HR-type resistance in K326^RTSW^ plants.

### 3.4. Role of NSm in HR Induction is Uncoupled from its Role in Plasmodesmata (PD) Targeting and Intercellular Movement

The NSm protein of TSWV is involved in the intracellular movement of TSWV ribonucleoproteins and intercellular movement of heterologous viruses [32,33,34,35]. However, whether the cell-to-cell movement function of NSm is coupled with its function in RTSW-mediated HR induction remains unknown. Li et al. [35] have identified domains and amino acid (aa) residues of the TSWV NSm protein necessary for tubule formation, intercellular movement, and symptom development using a heterologous *Tobacco mosaic virus* (TMV) expression system. Therefore, we investigated whether NSm mutants defective in tubule formation and cell-to-cell movement still retain the capacity to trigger HR against RTSW.

**Figure 4:**
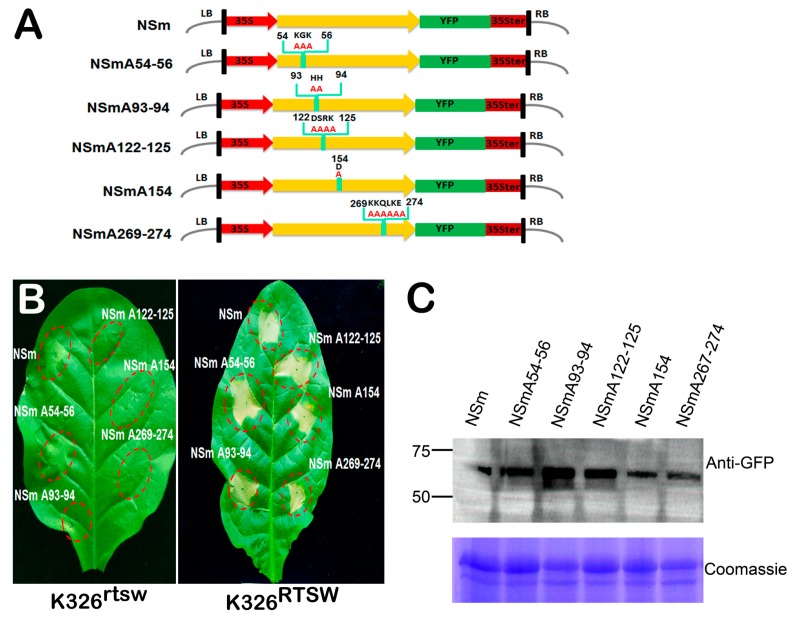
Analysis of the relationship between HR induction and movement functions of TSWV NSm. (**A**) Schematic representation of NSm-yellow fluorescent protein (YFP) constructs. YFP was fused to the C-terminal end of wild-type (WT) and mutant variants of NSm carrying alanine substitutions at amino acid (aa) residues 54–56 (NSmA54–56), 93 and 94 (NSmA93–94), 122–125 (NSmA122–125), 154 (NSmA154), or 269–274 (NSmA269–274). (**B**) Phenotypes of K326 leaves carrying the *RTSW* gene (K326^RTSW^) or lacking the *RTSW* gene (K326^rtsw^) after agroinfiltration with different constructs at 3 dpi. (**C**) Western blot analysis of WT or mutant NSm expression using anti-green fluorescent protein (GFP) antibody at 24 h post inoculation. Coomassie-stained gel was used as a loading control.

Our previously constructed five mutant forms of TSWV NSm carrying alanine substitutions at aa residues 54–56 (A54–56), 93 and 94 (A93–94), 122–125 (A122–125), 154 (A154), and 269–274 (A269–274), which were defective in both plasmodesmata (PD) targeting and tubule formation [25,35], fused with YFP (NSmA54–56-YFP, NSmA93–94-YFP, NSmA122–125-YFP, NSmA154-YFP, and NSmA269–274-YFP, respectively) were mobilized into *A. tumefaciens* and agroinfiltrated into *N. benthamiana* leaves (Figure 4A). Results of confocal laser scanning microscopy showed that none of these alanine substitution mutants localized at the PD, consistent with prior studies of these mutants [25,35]. Next, the ability of these NSm mutants defective in tubule formation to trigger HR was evaluated. Each NSm mutant was infiltrated into the leaves of K326^rtsw^ and K326^RTSW^ plants. Results showed that all NSm mutants triggered HR in K326^RTSW^ leaves (Figure 4B), similar to the wild-type NSm. Immunoblot analysis confirmed the comparable expression of wild-type and mutant NSm in infiltrated leaves (Figure 4C). Therefore, these results indicate that the function of NSm to target PD and traffic between cells is uncoupled from its function to trigger RTSW-mediated HR.

### 3.5. Key Amino Acids and a Conserved Motif Required for NSm-Sw-5b Interaction are Not Essential for RTSW Resistance

**Figure 5:**
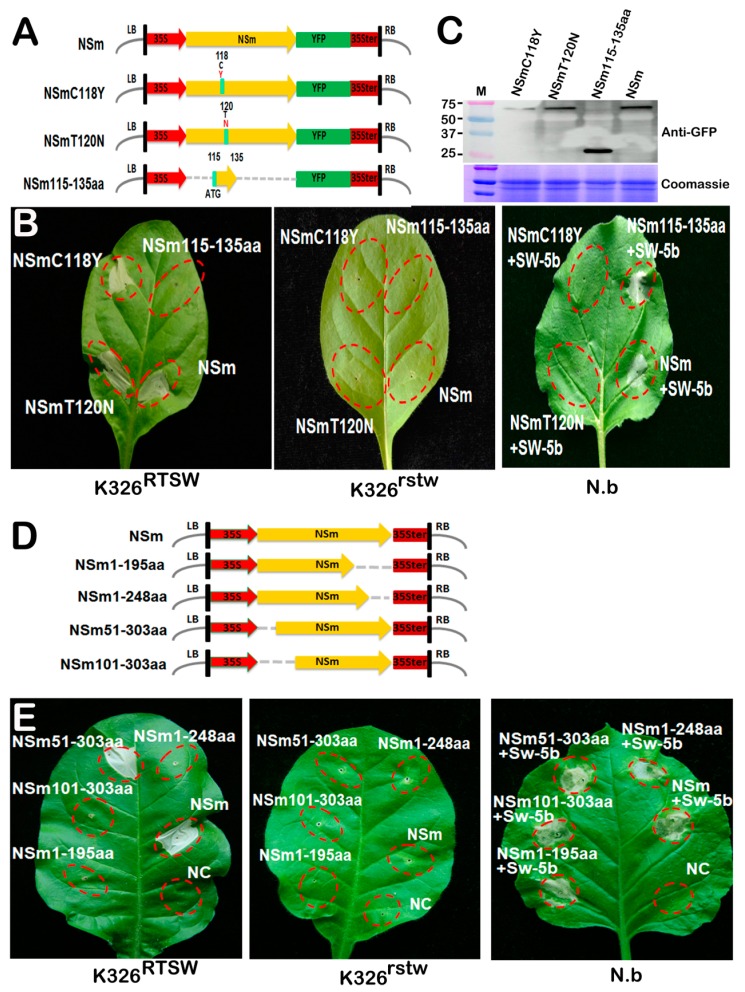
Effect of key amino acids and a conserved motif of NSm on RTSW-mediated induction of HR. (**A**) Schematic representation of NSm-YFP fusion constructs. NSm variants carrying aa substitutions at position 118 (NSmC118Y) or 120 (NSmT120N), or a conserved 21-aa peptide (115–135 aa) of NSm (NSm115–135aa), which is responsible for triggering Sw-5b-mediated HR, were fused with YFP. (**B**) Transient expression of NSm-YFP fusion constructs in leaves of susceptible (K326^rtsw^) and resistant (K326^RTSW^) *N. tabacum* plants. *Agrobacterium* cultures harboring the wild-type (WT) or mutant NSm construct alone or in conjunction with a culture harboring Sw-5b were infiltrated in *N. benthamiana* leaves. Photographs were taken at 3 dpi. (**C**) Immunoblot analysis of transient accumulation of WT or mutant NSm in tobacco leaves using anti-GFP antibody at 24 dpi. Coomassie-stained gel was used as a loading control. (**D**) Schematic representation of the expression cassettes of WT and truncated mutants of NSm protein. Yellow solid arrows depict the full-length NSm protein, and dotted lines indicate the deletion of approximately 50 or 100 aa from the N- or C-terminus of NSm. (**E**) Images of the K326^RTSW^, K326^rtsw^ and *N. benthamiana* (N.b) leaves infiltrated with truncated NSm proteins captured at 3 dpi. While all four truncated NSm proteins induced cell death in *N. benthamiana* leaf when co-infiltrated with Sw-5b, only NSm truncated for 51–303 aa induced HR symptoms in K326^RTSW^. Full-length NSm was used as a positive control, and the empty vector was used as a negative control (NC).

Both tomato Sw-5b and tobacco RTSW trigger HR through the recognition of TSWV NSm; this raises a question whether the same EAS of NSm is required for the elicitation/recognition. Previously, a naturally occurring aa mutation at position 118 or 120 in the TSWV NSm has been suggested in overcoming Sw-5b-mediated resistance in tomato [36]. Therefore, two NSm variants of TSWV, each carrying a single aa substitution at position 118 or 120 (NSmC118Y and NSmT120N, respectively), were constructed and agroinfiltrated into the leaves of K326^rtsw^ and K326^RTSW^ plants (Figure 5A). Both mutants induced necrosis in K326^RTSW^ leaves; however, none of the mutants were able to elicit HR in infiltrated K326^rtsw^ leaves or in *N. benthamiana* leaves co-infiltrated with the Sw-5b construct (Figure 5B). Western blot assay indicated that translational fusions of both NSm mutant proteins with YFP (NSmC118Y-YFP and NSmT120N-YFP) were expressed well in the infiltrated leaves (Figure 5C). These results suggest that the introduction of C118Y or T120N mutation in the NSm protein of TSWV does not affect the induction of cell death in plants carrying the RTSW gene.

Recently, it has been shown that the tomato Sw-5b gene confers broad-spectrum resistance against American-type orthotospoviruses by recognizing a conserved 21-aa sequence of NSm (NSm115–135aa) [16]. To determine if the RTSW gene is able to recognize the conserved 21-aa sequence of NSm and elicit HR, NSm115–135aa was fused to the N-terminal end of YFP (NSm115–135aa-YFP), and infiltrated into K326^rtsw^ and K326^RTSW^ leaves or co-infiltrated with Sw-5b into *N. benthamiana* leaves (Figure 5A). While the co-infiltration of NSm115–135aa-YFP and Sw-5b induced cell death in *N. benthamiana* leaves, the expression of NSm115–135aa-YFP in K326^RTSW^ or K326^rtsw^ leaves failed to trigger HR (Figure 5B). Immunoblot analysis confirmed the expression of NSm115–135aa-YFP in the infiltrated leaves (Figure 5C).

To further identify the sequence of NSm required for interaction with the RTSW gene, four mutant versions of the NSm protein truncated for approximately 50 or 100 aa at its N- or C-terminal end were generated (Figure 5D). In *N. benthamiana*, Sw-5b-mediated HR induced by the four mutant versions of NSm did not differ from that induced by the wild-type NSm (Figure 5E). By contrast, the NSm protein with the N-terminal 50-aa truncation (NSm51–303aa) induced HR to levels comparable to that induced by the full-length NSm (positive control) in K326^RTSW^ leaves. However, mutants with either 100-aa deletion from the N-terminus (NSm101–303aa) or deletions from the C-terminus (NSm1–248aa and NSm1–195aa) did not induce HR (Figure 5E). Thus, these results indicate that the 51–303 aa region of the NSm protein is important for the induction of RTSW-mediated cell death.

### 3.6. Application of the NSm Gene in Resistance Identification

**Figure 6:**
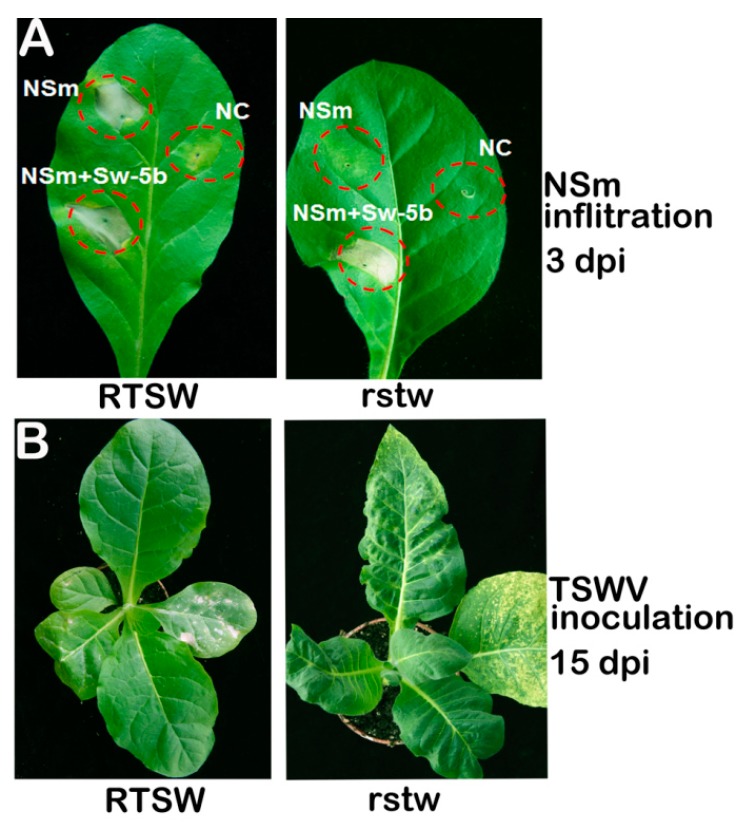
Development of an avirulent (Avr) gene-based diagnostic approach. (**A**) Necrotic response (HR lesions) was only observed in leaves of tobacco plants carrying the *RTSW* gene after transient expression of NSm. Co-infiltration of NSm with Sw-5b (NSm + Sw-5b) was used as a positive control, and agroinfiltration of empty vector was used as a negative control (NC) in each leaf. Photographs of leaves were captured at 3 dpi. (**B**) Typical symptom of TSWV in leaves of tobacco plants carrying the *RTSW* gene (K326^RTSW^) or lacking the *RTSW* gene (K326^rtsw^).

According to the ‘gene-for-gene’ model, an HR is induced by the specific interaction between the Avr gene product in the pathogen and the corresponding R gene product in the host plant [37]. In the present study, we identified NSm as the Avr gene of the TSWV; the NSm gene product is specifically recognized by the RTSW gene. To explore the application of the NSm gene in resistance determination, 120 plants of the BC5F1 population derived from the Polalta × K326 cross were randomly selected and surveyed by TSWV inoculation, NSm agroinfiltration, and genotyping using the SCAR marker S-AAC/CCC172 at the 4–6-leaf stage. A fully expanded leaf was detached and agroinfiltrated with NSm, NSm + Sw-5b (positive control), and empty vector (negative control). Agroinfiltrated leaves were incubated in a tray containing sterile distilled water, and cell death phenotype was recorded at 3 dpi (Figure 6A). Two other fully expanded leaves were mechanically inoculated with TSWV sap and symptoms were observed at 15 dpi (Figure 6B). Viral accumulation was analyzed twice using ELISA at 15 and 30 dpi. To genotype the plants using the SCAR marker S-AAC/CCC172, DNA was isolated from a new emerging leaf of each plant and subjected to PCR. The results of NSm agroinfiltration and SCAR marker analysis, indicating phenotypic and genotypic segregation, respectively, were similar; 58 plants were positive and 62 were negative for the RTSW gene (Table S1). Thus, the ratio approached 1:1, as expected for the segregation of a single dominant gene. However, results of ELISA indicated that 65 plants were resistant and 55 were susceptible to TSWV (Table S1). Thus, ELISA results were different from NSm agroinfiltration and SCAR marker-based genotyping results for 7 of the 65 plants. These seven plants were re-inoculated with TSWV, and three plants were infected. The remaining four F1 plants were selfed, and their progeny was tested to determine the TSWV resistance phenotype. Each F2 population contained 10 plants, all of which showed a susceptible phenotype in TSWV ELISA (Table S1). This suggests that TSWV sap inoculation resulted in seven false positives. Thus, our results indicate that the Avr NSm gene is more efficient than mechanical inoculation of TSWV sap for the identification of genotypes resistant to TSWV.

## 4. Discussion

The RTSW gene in tobacco confers resistance to TSWV [22,23]. In this study, we showed that the RTSW resistance gene protects the host plant against TSWV infection by inducing HR, associated with rapid cell death on inoculated leaves as well as significant expression of defense-related genes. To identify the TSWV Avr determinants, we used agroinfiltration to transiently express genes encoding the Gn, Gc, NSm, N, and NSs proteins of TSWV in *Nicotiana* species. Among these five proteins, only the movement protein (MP) NSm elicited HR in *N. alata*, Polalta, and K326^RTSW^ plants carrying the RTSW gene. To verify the role of NSm as an Avr determinant, the *NSm* gene was expressed in PVX vectors and agroinfiltrated into leaves of *Nicotiana* species. Unlike the empty PVX vector, which induced systemic infection in all test plants, PVX-NSm elicited HR-type cell death, thus preventing systemic infection and arresting the virus in the inoculated K326^RTSW^ leaf. These findings unequivocally indicate that the NSm protein of TSWV is the Avr determinant of RTSW-based TSWV resistance.

The NSm protein has been well characterized as a typical plant virus MP, and has been associated with intracellular localization close to PD, the formation of tubule structures [34,35], and interactions with a host trafficking protein [38,39]. Five alanine substitution mutants of TSWV NSm (A54–56, A93–94, A122–125, A154, and A269–274) defective in PD localization, tubule formation, and cell-to-cell movement were generated. Surprisingly, all of the NSm mutants retained their ability to induce HR in tobacco leaves carrying the RTSW gene, similar to the wild-type NSm. Therefore, our results clearly demonstrate that the function of NSm to target PD and to traffic between cells is not linked to its function to trigger RTSW-mediated HR. This observation is consistent with a variety of plant viruses. The helicase domain (p50) of the TMV replicase protein induces N-mediated defense response in tobacco; however, the mutation that abolishes the ATPase activity of p50 does not affect HR induction [40]. Hu et al. have shown that the conserved GDD (Gly-Asp-Asp) motif in the 2a replicase protein of the *Cucumber mosaic virus* (CMV) is essential for CMV replication but not for HR induction [41]. Similarly, the TSWV NSs protein, which acts as a suppressor of RNA silencing, also represents the Avr factor of the Tsw resistance gene in pepper. However, the activity of NSs as an RNA silencing suppressor is distinct from its activity as an Avr factor [42]. Recently, it has been suggested that the intercellular trafficking of NSm is uncoupled from its function in HR induction; this has become evident from the analysis of NSm mutants defective in targeting PD and cell-to-cell movement but capable of inducing Sw-5b-mediated HR [25,43]. Our results obtained in this study and previous studies support the hypothesis that rather than recognizing the domains involved in virus replication, movement, or gene-silencing suppression, R protein may have first evolved to recognize the most conserved domains of viral effector proteins [25]. This assumption is strongly supported by the most recent study on Sw-5b and NSm proteins [16]; Zhu et al. show that Sw-5b confers broad-spectrum resistance against American-type orthotospoviruses by recognizing a small 21-aa (NSm115–135aa) conserved PAMP-like region in the NSm protein. Our results indicated the importance of the 51–303 aa region of NSm in triggering RTSW-mediated resistance. Further research is needed to confirm whether similar small core regions exist within 51–303 aa of NSm for the recognition of RTSW.

It is noteworthy to mention that the TSWV NSm has recently been demonstrated as the Avr determinant of the Sw-5b gene in tomato [17,18]. Numerous studies have revealed that homologous genes in different *Solanum* species exhibit conserved biological functions. The NSm protein triggers HR in tobacco and tomato, as shown in this study and previous studies, suggesting that these species share an orthologous R protein, which recognizes the NSm protein. Alternatively, it is also possible that the NSm protein is targeted by divergent R proteins in tobacco and tomato. Several lines of evidence indicate that different genes, which confer resistance to the same Avr protein of the same virus, recognize different EASs. The MP of *Tomato mosaic virus* (ToMV) elicits both Tm-2 and Tm-2^2^; however, the elicitor active domains (EADs) map to the N- and C-terminus of MP, respectively. The *Rx* gene confers resistance to PVX through its interaction with conserved amino acids 121–127 of PVX coat protein (CP), whereas the *Nx* gene confers resistance to PVX through the recognition of aa 62–78 of PVX-CP. In this study, we also analyzed whether the critical aa residues Y118 and N120 or the conserved 21-aa peptide of NSm (115–135 aa), which has been proposed to be responsible for Sw-5b-mediated resistance, were necessary to elicit HR in tobacco plants carrying the *RTSW* gene. Interestingly, opposite patterns of these mutants were observed in RTSW-harboring tobacco and Sw-5b co-infiltrated *N. benthamiana* leaves in this study. Furthermore, different patterns were observed with the truncated versions of the NSm protein to trigger HR against RTSW and Sw-5b. Unlike the 100-aa truncation of NSm at both ends, which induced HR against Sw-5b [16], only the 50-aa truncation at the N-terminal end of NSm did not affect its avirulence to trigger RTSW-mediated HR. These findings suggest that although RTSW and Sw-5b behave as single dominant genes conferring resistance against TSWV and recognize the same Avr protein (NSm), RTSW is likely not an ortholog of Sw-5b, since it recognizes different EADs.

A precise disease resistance test is a prerequisite for the identification of segregation for virus resistance in a population, and consequently, for the fine mapping and cloning of resistance genes. Generally, a disease resistance test involves the inoculation of plants with infectious clones, insect vectors, or pathogen-containing sap. However, for TSWV, the first two approaches have proven to be challenging because of the lack of an infectious clone and difficult management of thrips. Several factors influence the rate of mechanical transmission of TSWV using infected plant sap, such as the plant growth environment, plant growth stage, and the source of inoculum, antioxidants, and abrasives [44]. The process of TSWV sap inoculation is complicated, time consuming, and labor intensive, and therefore inefficient. Moreover, the identification of disease resistant phenotypes using mechanical sap inoculation is not accurate and results in false positives. Therefore, there is an urgent need for the development of a rapid disease resistance test that overcomes the limitations of sap inoculation and greatly improves the efficiency and accuracy of inoculation of test plants. In the current study, we explored the use of an *Avr* gene-based diagnostic tool for determining the presence and effectiveness of R genes. It is reasonable to speculate that the necrosis of NSm-infiltrated leaf area was due to the presence of the RTSW gene. To validate our hypothesis, an expected 1:1 ratio of resistant:susceptible plants in the F1 population was obtained and used for genetic analysis. In contrast to the seven false positives obtained using TSWV sap inoculation, results of the Avr gene-based diagnostic test were consistent with those of SCAR marker-based genotyping. Furthermore, while the TSWV sap inoculation approach took approximately a month for analysis, the Avr gene-based diagnostic took only 2–3 days for inoculant preparation and phenotype monitoring. Taken together, we conclude that the Avr gene-based diagnostic tool is a precise and RTSW-specific assessment method. This method can be used not only to determine the presence of the RTSW gene but also to test the effectiveness of target RTSW genes in tobacco.

## Supplementary Materials

The following are available online at http://www.mdpi.com/1999-4915/10/11/660/s1, Table S1: Evaluation of resistance to *Tomato spotted wilt virus* (TSWV) by virus inoculation, SCAR marker analysis, and *Avr*-based diagnosis.

## Appendix A

**Table A1:** Primers used in this study.

Primer Name	Primer Sequence (5′→3′)
Primers used for the construction of expression vectors	
NSmBamHIF	CGGGATCCATGTTGACTTTTTTTGGTA
NSmBamHIR	CGGGATCCTATCTCATCAAAAGATAACT
GnBamHIF	CCCGGATCCATGAGAATTCTAAAACTA
GnBamHIR	CCCGGATCCTCATTCCATGCTAGTCCACT
GcBamHIF	CGGGATCCATGTGGTTCCATCTAATAGTGAAC
GcBamHIR	CGGGATCCTCAGACAAGGTGAGAGAAATC
NSsBamHIF	CGGGATCCATGTCTTCAAGTGTTTATGAG
NSsBamHIR	CGGGATCCTTATTTTGATCCTGAAGCATATG
NBamHIF	CGGGATCCATGTCTAAGGTTAAGCTCACT
NBamHIR	CGGGATCCTTAAGCAAGTTCTGCAAGT
NSmCalIF	GGCATCGATATGTTGACTTTTTTTGGTA
NSmSalIR	GGCGTCGACCTATATCTCATCAAAAGATAAC
Primers used for qRT-PCR	
PR1qRTF	AGCTCGTGCAGATGTAGGTGTAGA
PR1qRTR	TTCGCCGTATTGACCATGAGAATGT
HSR203JqRTF	CGCAATCATCGTCTCCGTCTTCC
HSR203JqRTR	CTCCGATAAGACCGCACGAACC
GAPDHF	GCAGTGAACGACCCATTTATCTC
GAPDHR	AACCTTCTTGGCACCACCCT
Primers used for genotyping	
S-AAC/CCC172F	AGCTTCTTTTCTCTTTCCATTTTT
S-AAC/CCC172R	CAGAAGAAAAACTGCTGGAGCTAT
Actin1000F	ATGGGCCAGAAGGATGCTTATG
Actin1000R	CTGCTGGAATGTGCTGAGAGAG
